# Frequência e Motivos para a não Administração e Suspensão de Medicamentos durante um Evento de Síndrome Coronariana Aguda. Estudo ERICO

**DOI:** 10.36660/abc.20190317

**Published:** 2020-11-01

**Authors:** Rafael C. O. Santos, Isabela M. Bensenor, Alessandra C. Goulart, Paulo A. Lotufo, Itamar S. Santos

**Affiliations:** 1 Universidade de São Paulo Hospital Universitário da USP Centro de Pesquisa Clínica e Epidemiológica São PauloSP Brasil Universidade de São Paulo - Centro de Pesquisa Clínica e Epidemiológica do Hospital Universitário da USP, São Paulo, SP - Brasil; 2 Universidade de São Paulo Faculdade de Medicina da USP Departamento de Clínica Médica São PauloSP Brasil Universidade de São Paulo - Departamento de Clínica Médica da Faculdade de Medicina da USP, São Paulo, SP - Brasil

**Keywords:** Síndrome Coronariana Aguda/mortalidade, Suspensão do Tratamento/tratamento farmacológico, Morbidade, Atenção à Saúde

## Abstract

**Fundamentos:**

Poucos estudos discutiram causas para o subtratamento medicamentoso na SCA.

**Objetivos:**

Avaliar a não-administração e suspensão de medicamentos durante o tratamento intra-hospitalar da SCA na Estratégia de Registro de Síndrome Coronariana Aguda (estudo ERICO).

**Métodos:**

Analisamos prontuários de 563 participantes ERICO para avaliar a frequência e motivos da não administração e/ou suspensão de medicamentos. Construímos modelos de regressão logística para avaliar se sexo, idade ≥65 anos, nível educacional ou subtipo de SCA estavam associados com (a) não administração de ≥1 medicamentos; e (b) não administração ou suspensão de ≥1 medicamentos. O nível de significância foi 5%.

**Resultados:**

A amostra é composta por 58,1% de homens e com idade mediana de 62 anos. Em 183 (32,5%) participantes ≥1 medicamentos não foram administrados e 288 (51,2%) apresentaram ≥1 medicamentos não administrados ou suspensos. As causas mais frequentes foram risco de sangramento (aspirina, clopidogrel e heparina), insuficiência cardíaca (betabloqueadores) e hipotensão (inibidores da enzima conversora da angiotensina e bloqueadores dos receptores da angiotensina). Indivíduos com idade ≥65 anos (razão de chances [RC]:1,51; intervalo de confiança de 95% [IC95%]:1,05-2,19) e com angina instável (RC:1,72; IC95%:1,07-2,75) tiveram maior chance de não-administração. Considerando apenas pacientes com infarto do miocárdio, idade ≥65 anos foi associada tanto à não administração quanto à não administração ou suspensão.

**Conclusões:**

A não administração ou suspensão de ≥1 medicamento não foi rara no estudo ERICO. Indivíduos com idade ≥65 anos ou com angina instável tiveram maior chance de não administração e podem ser subtratados nesse cenário.

## Introdução

A doença arterial coronária (DAC) continua a ser a principal causa de mortalidade e esperança de vida corrigida pela incapacidade em todo o mundo, inclusive no Brasil.^1^^–^^4^ O tratamento adequado e no momento certo pode reduzir a morbidade e mortalidade.^5^ Há evidências de que a qualidade do tratamento farmacológico durante a fase hospitalar de um caso de síndrome coronariana aguda (SCA), definido pela administração de medicamentos orientados pelas diretrizes, está associada à sobrevivência à internação hospitalar^6^ e à sobrevivência seis meses após alta hospitalar.^7^

No maior estudo brasileiro que relata a frequência da prescrição de medicamentos orientada por diretrizes em pacientes de SCA hospitalizados até agora, Wang et al.,^8^ analisaram dados de 2.453 indivíduos com SCA de 65 hospitais brasileiros (aproximadamente 90% de hospitais terciários) no estudo do Registro *Acute Coronary Care Evaluation of Practice* (ACCEPT) de agosto de 2010 a dezembro de 2011. Entre os medicamentos analisados em seu estudo, a aspirina foi o medicamento mais comumente receitado nas primeiras 24 horas (97,6%). As estatinas também foram prescritas com alta frequência (90,6%).

Poucos estudos discutiram os motivos para o subtratamento da SCA. Isso ganha importância especial à medida que a idade dos pacientes de SCA aumenta. Efeitos adversos e contraindicações são mais frequentes^9^ em indivíduos mais velhos, contribuindo para morbidade e mortalidade associadas mais altas.^10^^,^^11^ Marino et al.,^12^ avaliaram 583 indivíduos diagnosticados com SCA em seis hospitais de emergência em Montes Claros. Nas primeiras 24 horas de tratamento, o uso de medicamentos para o tratamento de SCA variou de 63,8% (heparinas) a 96,6% (aspirina). Dos 181 pacientes (31,0% da amostra) que não receberam betabloqueadores dentro de 24 horas, 39 (21,5%) apresentaram contraindicações identificáveis. Não foram relatadas outras descrições das causas para o subtratamento durante as primeiras 24 horas.

O presente artigo tem o objetivo de analisar os dados de eventos de SCA, o que levou ao cadastro de 563 participantes do Estudo de Estratégia Registro de Insuficiência Coronariana (ERICO), um estudo prospectivo que está em andamento no Hospital Universitário da Universidade de São Paulo (HU-USP). Nossa equipe buscou determinar a frequência de uso, juntamente com os motivos para não administração e suspensão dos medicamentos usados durante o tratamento hospitalar de um evento de SCA e seus fatores associados.

## Métodos

### Desenho do Estudo ERICO

O desenho do estudo ERICO foi descrito em detalhe em outro local.^13^^,^^14^ Resumidamente, ERICO é um estudo prospectivo de observação de 1.085 indivíduos internados no HU-USP devido a um evento de SCA entre fevereiro de 2009 e dezembro de 2013. O HU-USP é um hospital comunitário no Butantã, um bairro da cidade de São Paulo, com uma população aproximada de 428.000 habitantes, em 2010, e desigualdades sociais pronunciadas.

Para participar do estudo ERICO, os participantes precisavam atender a critérios diagnósticos de infarto do miocárdio com supradesnivelamento de ST (*ST-elevation myocardial infarction* - STEMI), infarto do miocárdio sem supradesnivelamento de ST (*Non ST-elevation myocardial infarction* - NSTEMI) ou angina instável (AI). Para o diagnóstico do infarto do miocárdio (IM), ambos os critérios a seguir devem estar presentes: (I) Sintomas consistentes com isquemia cardíaca nas primeiras 24 horas de internação hospitalar e (II) Níveis de troponina I acima do 99º percentil, com um coeficiente de variação específico do teste <10%. O diagnóstico de STEMI exige ambos os seguintes critérios: (I) Critérios para diagnóstico de IM e (II) Um dos seguintes: (a) supradesnivelamento persistente do segmento ST ≥ 1 mm em duas derivações eletrocardiográficas contíguas ou (b) a presença de um bloqueio do ramo esquerdo novo ou presumivelmente novo. Para o diagnóstico de NSTEMI, os participantes devem apresentar: (I) Critérios para diagnóstico de IM e (II) Ausência de diagnóstico de STEMI. Para diagnóstico de AI, todos os três critérios a seguir devem ser atendidos: (I) Sintomas consistentes com isquemia cardíaca 24 horas antes da internação hospitalar, (II) Ausência de critérios de IM, e (III) Pelo menos um dos seguintes: (a) histórico de doença arterial coronária; (b) teste positivo de estratificação de doença coronária (invasivo ou não invasivo); (c) alterações do segmento ST transitórias ≥ 0,5 mm em duas derivações contíguas, novas inversões de onda T de ≥ 1 mm e/ou pseudonormalização de ondas T invertidas previamente; (d) troponina I > 0,4 ng/ml; ou (e) concordância no diagnóstico de dois médicos independentes. A síndrome coronariana aguda sem supradesnivelamento de ST (SCASST) é um termo comum que abrange NSTEMI e AI.

Na linha de base, entrevistadores treinados obtiveram dados sobre aspectos sociodemográficos e fatores de risco cardiovascular, bem como sobre medicamentos usados anteriormente. Durante a fase de internação hospitalar, todos os sujeitos foram tratados a critério da equipe do hospital, com procedimentos padrão, e sem influência do protocolo do estudo. O acompanhamento de longo prazo continua sendo feito, com contatos telefônicos anuais.

### Desenho do estudo ERICO-APS

O presente trabalho é uma análise de um estudo ERICO (Estratégia Registro de Insuficiência Coronariana - Atenção Primária à Saúde; estudo ERICO-APS). Maiores detalhes sobre o estudo ERICO-APS podem ser encontrados em uma publicação anterior.^15^ O ERICO-APS tem o objetivo de estudar determinantes da qualidade da atenção de saúde e a mortalidade, com foco especial na unidade do primeiro contato (atenção primária ou hospital) durante o evento inicial de SCA. O ERICO-APS é composto por 130 participantes para os quais uma unidade de atenção primária de saúde foi a unidade onde aconteceu o primeiro contato durante o evento inicial, e 700 participantes que vieram diretamente ao hospital, todos cadastrados no estudo principal de fevereiro de 2009 a dezembro de 2012.

### Amostra do estudo

Em nossas análises, os participantes do ERICO-APS que vieram diretamente ao hospital foram considerados elegíveis. Esse estudo excluiu 44 (6,3%) participantes cujos prontuários médicos não puderam ser obtidos, e 93 (13,3%) cujos prontuários médicos estavam incompletos (por exemplo, devido a transferência para outros hospitais). Nossa amostra final foi composta de 563 participantes do ERICO-APS.

### Variáveis do estudo

Os diagnósticos de hipertensão, diabetes, dislipidemia, e doença arterial coronária (DAC) prévia foram definidos por autorrelato. O tabagismo foi classificado em nunca fumou, ex-fumante e fumante. O nível de escolaridade foi autorrelatado, e classificado em sem educação formal, de 1 a 7 anos de educação formal, e ≥ 8 anos de educação formal. Em algumas das análises, a idade foi categorizada usando-se uma linha de corte de 65 anos.

Os prontuários médicos e as prescrições foram revisados para se analisar a frequência de administração, os motivos para a não administração, e os motivos para a suspensão dos seguintes medicamentos: aspirina, clopidogrel, heparinas, betabloqueadores, e inibidores da enzima conversora da angiotensina e bloqueadores de receptores da angiotensina (IECA/BRA). A frequência da administração de estatinas, nitratos, e morfina também foi analisada.

“Não administração” foi definida como a não prescrição de medicamentos desde a admissão até a alta hospitalar. “Suspensão” foi definida como a retirada de medicamentos prescritos inicialmente durante o período de internação hospitalar. Uma exceção foi a retirada da prescrição de heparina após o oitavo dia de internação hospitalar.^16^ Os motivos foram separados por classe farmacológica: (a) aspirina: alergia, sangramento ou risco de sangramento, e cirurgia de revascularização; (b) clopidogrel: sangramento ou risco de sangramento e *bypass* de artéria coronária; (c) heparina: sangramento ou risco de sangramento, cirurgia de revascularização, síndrome coronariana aguda de baixo risco, e angiografia coronária; (d) betabloqueadores: broncoespasmo, bradicardia, choque/hipotensão, insuficiência cardíaca descompensada, e testes não invasivos de isquemia; e (e) IECA/BRA: insuficiência renal crônica (IRC), choque/hipotensão, insuficiência renal aguda (IRA), e hipercalemia.

Esses motivos estão descritos na tabela suplementar 1, juntamente com os medicamentos mais comumente prescritos para cada classe farmacológica. A não administração (ou suspensão) de qualquer medicamento foi definida como a não administração (ou suspensão) de um ou mais dos seguintes medicamentos: aspirina, clopidogrel, heparina, betabloqueadores, estatinas, e/ou inibidores ECA/BRA.

Quando o motivo da não administração ou da suspensão do medicamento havia sido anotado nos prontuários médicos, essa informação era recuperada e classificada de acordo com esse motivo explícito. Quando os motivos da não administração ou da suspensão do medicamento não eram explícitos, um médico e um farmacêutico do estudo analisavam o prontuário médico para verificar se algum dos motivos descritos estavam implícitos. Portanto, os motivos da não administração ou da suspensão foram classificados como “não descritos”, “implícitos”, ou “explícitos”.

O status vital foi avaliado por entrevista telefônica 30 dias após o evento inicial, de acordo com o protocolo do estudo ERICO.^14^^,^^17^ Os registros oficiais de mortes foram obtidos com a colaboração de registros de óbitos municipais e estaduais sempre que se verificou que o participante havia falecido, ou se o paciente não pudesse ser contatado naquele momento.

### Considerações éticas

O protocolo do estudo estava de acordo com a Declaração de Helsinki. O comitê de análise institucional do hospital aprovou o protocolo de pesquisa (Aprovação do comitê de ética 866/08). Foi obtido o consentimento informado por escrito de todos os pacientes de SCA internados no hospital que concordaram em participar do estudo, e cada sujeito recebeu uma cópia do formulário de consentimento informado.

### Análise estatística

As variáveis categóricas são apresentadas em contagem absoluta e em proporções, e comparadas utilizando-se testes qui-quadrados. Devido a sua distribuição não normal (avaliada por gráficos de densidade e pelo teste de Shapiro-Wilk), a idade é apresentada como média e faixa interquartil e comparada entre os grupos, utilizando-se o teste de Kruskal-Wallis. Este estudo também realizou comparações por pares (com correção de Holm) da distribuição das idades em grupos de STEMI, NSTEMI e AI. Modelos de regressão logística bruta ou múltipla foram construídos para analisar sexo, idade ≥ 65 anos, nível de escolaridade, ou subtipo de SCA foram associados com (a) a não administração de qualquer medicamento e (b) a não administração ou suspensão de qualquer medicamento. Como análise de sensibilidade, esses modelos foram repetidos: (a) excluindo a não administração/suspensão devido a angioplastia coronária transluminal percutânea (ACTP) e/ou enxerto de *bypass* da artéria coronária (*coronary artery bypass graft* CABG) agendados e (b) excluindo os casos com angina instável, já que alguns medicamentos podem não ter sido prescritos devido à SCA de baixo risco. As curvas de Kaplan-Meier e o teste de *long-rank* foram usados para determinar se a sobrevivência após 30 dias estava associada com ≥ 1 medicamentos não administrados ou suspensos. O nível de significância foi definido em 5%. O software R, versão 3.2.0, foi utilizado na realização dessas análises.^18^

## Resultados

A tabela 1 mostra as características de linha de base da amostra do estudo, de acordo com subtipo de SCA. A amostra desse estudo teve uma predominância de sujeitos do sexo masculino (n=327; 58,1%), com uma média de 62 anos de idade. Indivíduos com STEMI tinham idade mais baixa em comparação com os indivíduos com NSTEMI (p=0,002) e AI (p=0,024). A distribuição de idades dos participantes com NSTEMI e AI não é significativamente diferente (p=0,35). Hipertensão (n=421; 76,5%) e sedentarismo (n=369; 70,3%) foram os fatores de risco cardiovascular mais frequentes na amostra. Apenas 150 (29,1%) dos participantes tinham um diagnóstico prévio de DAC antes do evento de SCA que havia levado ao cadastro no estudo ERICO.

**Tabela 1:** Características da linha de base da amostra do estudo

		STEMI (N=162)	NSTEMI (N=232)	AI (N=169)	Total (N=563)
Idade (anos; média [FIQ])		59,0 [50,0 - 68,0]	64,0 [53,8 - 74,0]	62,0 [53,0 - 73,0]	62,0 [52,0 - 72,0]
Sexo masculino		106 (65,4%)	140 (60,3%)	81 (47,9%)	327 (58,1%)
Nível de escolaridade					
	Sem educação formal	16 (9,9%)	24 (10,3%)	22 (13,0%)	62 (36,7%)
	1 a 7 anos	69 (42,9%)	107 (46,1%)	62 (11,0%)	238 (42,3%)
	≥ 8 anos	76 (47,2%)	101 (43,5%)	85 (50,3%)	262 (46,6%)
Hipertensão		101 (64,3%)	174 (76,0%)	146 (89,0%)	421 (76,5%)
Diabetes		49 (31,4%)	99 (42,9%)	67 (41,4%)	215 (39,2%)
Dislipidemia		66 (50,0%)	113 (53,3%)	83 (57,2%)	262 (53,6%)
Sedentarismo		98 (66,2%)	156 (70,6%)	115 (73,7%)	369 (70,3%)
Tabagismo					
Nunca fumou		37 (23,7%)	69 (31,7%)	60 (38,5%)	166 (31,3%)
Ex-fumante		57 (36,5%)	81 (37,2%)	62 (39,7%)	200 (37,7%)
Fumante		62 (39,7%)	68 (31,2%)	34 (21,8%)	164 (30,9%)
DAC Prévia		25 (16,9%)	50 (23,3%)	75 (49,3%)	150 (29,1%)

*FIQ: faixa interquartil; STEMI: infarto do miocárdio com supradesnivelamento de ST; NSTEMI: infarto do miocárdio sem supradesnivelamento de ST; AI: angina instável; DAC: doença arterial coronária.*

A tabela 2 mostra a frequência da administração de aspirina, clopidogrel, heparinas, estatinas, betabloqueadores, IECA ou BRA, nitratos, e morfina durante o tratamento hospitalar. Considerando os principais medicamentos no tratamento da SCA (aspirina, clopidogrel, heparina, betabloqueadores, estatinas, e/ou inibidores de ECA/BRA), este estudo identificou 183 (32,5%) dos participantes aos quais um ou mais medicamentos não foram administrados. O uso de nitrato foi semelhante, de acordo com o subtipo de SCA (p=0,32) e, conforme esperado, a administração de morfina foi mais frequente nos participantes com diagnóstico de STEMI (p<0,001). Em 288 (51,2%) participantes, este estudo observou a não administração ou a suspensão de um ou mais dos principais medicamentos durante o tratamento hospitalar.

**Tabela 2:** Administração de medicamentos orientados por diretrizes durante internação hospitalar

Medicamento		STEMI	NSTEMI	AI	Total
Aspirina		158 (97,5%)	229 (98,7%)	165 (97,6%)	552 (98,0%)
Clopidogrel		159 (98,1%)	226 (97,4%)	158 (93,5%)	543 (96,4%)
Heparina		153 (94,4%)	228 (98,3%)	160 (94,7%)	541 (96,1%)
Estatinas		152 (93,8%)	217 (93,5%)	147 (87,0%)	516 (91,7%)
Betabloqueadores		138 (85,2%)	194 (83,6%)	142 (84,0%)	474 (84,2%)
IECA/BRA		136 (84,0%)	201 (86,6%)	132 (78,1%)	469 (83,3%)
Nitrato		95 (58,6%)	119 (51,3%)	95 (56,2%)	309 (54,9%)
Morfina		37 (22,8%)	30 (12,9%)	9 (5,3%)	76 (13,5%)

STEMI: infarto do miocárdio com supradesnivelamento de ST; NSTEMI: infarto do miocárdio sem supradesnivelamento de ST; AI: angina instável; IECA/BRA: inibidores da enzima conversora da angiotensina ou bloqueadores de receptores da angiotensina.

A tabela 3 apresenta os motivos para a não administração ou suspensão de aspirina, clopidogrel, heparina, betabloqueadores e IECA/BRA. Observou-se que a não administração de aspirina, clopidogrel, e heparina é rara, geralmente associada a um maior risco de sangramento. Os motivos mais frequentes para a não administração de betabloqueadores foram a insuficiência cardíaca descompensada, e choque/hipotensão. A insuficiência cardíaca também foi o motivo mais frequente para a suspensão de betabloqueadores. Choque/hipotensão foi o motivo mais frequente para a não administração e suspensão de IECA/BRA. A tabela suplementar 2 relata as frequências da presença dos motivos da não administração/suspensão dos medicamentos nos prontuários médicos. Observou-se que os motivos da não administração não foram descritos nos prontuários médicos em 64,0% dos casos, e os motivos para a suspensão não foram descritos em 26,4%.

**Tabela 3:** Causas da não administração ou suspensão de medicamentos na amostra

Medicamento	Causa	Não administração	Suspensão
Aspirina	Alergia	4	0
	Sangramento ou risco de sangramento	1	5
	Enxerto de *bypass* da artéria coronária	0	5
	Total	5	10
Clopidogrel	Sangramento ou risco de sangramento	1	15
	Enxerto de *bypass* da artéria coronária	1	2
	Angiografia coronária	0	22
	Total	2	39
Heparina	Sangramento ou risco de sangramento	2	7
	Enxerto de *bypass* da artéria coronária	0	4
	Angiografia coronária	0	35
	Síndrome coronariana aguda de baixo risco	2	0
	Total	4	46
Betabloqueadores	Insuficiência cardíaca descompensada	16	11
	Broncoespasmo	14	5
	Choque/hipotensão	14	5
	Bradicardia	6	4
	Teste não invasivo de isquemia	0	1
IECA/BRA	Choque/hipotensão	14	9
	Insuficiência renal crônica	6	0
	Hipercalemia	3	5
	Insuficiência renal aguda	1	7
	Total	24	21

*IECA/BRA: inibidores da enzima conversora da angiotensina ou bloqueadores de receptores da angiotensina*

A tabela 4 mostra a razão de chances (a partir de modelos múltiplos) para a não administração e não administração/suspensão de um ou mais medicamentos (aspirina, clopidogrel, heparina, estatinas, e/ou inibidores de ECA/BRA) associados a idade, sexo, nível de escolaridade, e subtipo de SCA. A análise de toda a amostra revelou que indivíduos com 65 anos de idade ou mais (p=0,027) e indivíduos com angina instável (p=0,025) apresentaram uma probabilidade mais alta de não administração de um ou mais medicamentos. Quando os indivíduos com angina instável foram excluídos, ter ≥ 65 anos de idade foi associado com a não administração (p=0,023) ou a não administração/suspensão (p=0,035) de um ou mais medicamentos. Nessa subamostra, os indivíduos com STEMI ou NSTEMI apresentaram probabilidade semelhante de não administrador (p=0,73) ou a não administração/suspensão (p=0,85) de um ou mais medicamentos.

**Tabela 4:** Razão de chances (95% IC) de vários modelos para a associação entre não administração e não administração e suspensão com idade, sexo, nível de escolaridade, e subtipo de SCA

		Todos os tipos de SCA		Excluindo participantes com AI	
		Não administração	Não administração ou suspensão	Não administração	Não administração ou suspensão
Sexo masculino		0,96 (0,67 - 1,39)	0,88 (0,62 - 1,24)	0,98 (0,62 - 1,55)	0,93 (0,61 - 1,41)
Idade ≥ 65 anos		**1,51 (1,05 - 2,19)**	1,36 (0,96 - 1,92)	**1,69 (1,07 - 2,67)**	**1,57 (1,03 - 2,40)**
Nível de escolaridade					
	Sem educação formal	0,58 (0,31 - 1,11)	0,58 (0,32 - 1,03)	0,58 (0,25 - 1,33)	0,55 (0,26 - 1,13)
	1 a 7 anos	0,90 (0,61 - 1,31)	1,10 (0,77 - 1,58)	0,95 (0,60 - 1,51)	1,16 (0,76 - 1,76)
	≥ 8 anos	1.0 (Referência)	1.0 (Referência)	1.0 (Referência)	1.0 (Referência)
Subtipo de SCA					
	STEMI	1.0 (Referência)	1.0 (Referência)	1.0 (Referência)	1.0 (Referência)
	NSTEMI	1,10 (0,70 - 1,73)	0,98 (0,65 - 1,48)	1,08 (0,69 - 1,71) -	0,96 (0,64 - 1,45) -
	AI	**1,72 (1,07 - 2,75)**	1,23 (0,79 - 1,91)

*p<0,05 em negrito. SCA: Síndrome coronariana aguda; STEMI; infarto do miocárdio com supradesnivelamento de ST; NSTEMI: infarto do miocárdio sem supradesnivelamento de ST; AI: angina instável.*

Análises de sensibilidade, considerando que o fato de participantes terem ACTP e CABG agendados não se qualificava como um motivo para a não administração e/ou a suspensão de clopidogrel e heparinas (tabela suplementar 3), levaram a conclusões semelhantes, exceto por uma associação significativa entre ter ≥ 65 anos de idade e a não administração/suspensão de um ou mais medicamentos (razão de chances: 1,44; 95% IC: 1,02 – 2,04). As tabelas suplementares 4 e 5 mostram os resultados obtidos dos modelos brutos.

**Figura 1:**
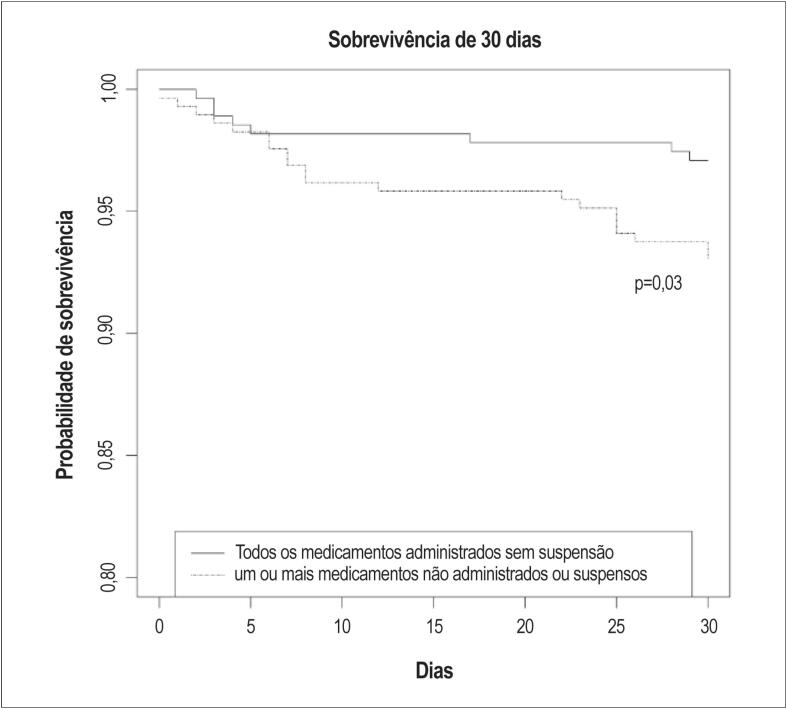
Sobrevivência após 30 dias para indivíduos que (a) tiveram todos os medicamentos administrados sem suspensão, e (b) tiveram um ou mais medicamentos não administrados ou suspensos.

Após 30 dias, oito (2,9%) indivíduos aos quais todos os medicamentos foram administrados sem suspensão, e 20 (6,9%) indivíduos com um ou mais medicamentos não administrados ou suspensos haviam morrido (Figura 1). A sobrevivência após 30-dias foi significativamente associada à presença de um ou mais medicamentos não administrados ou suspensos (p=0,03).

## Discussão

O presente estudo observou que, durante o tratamento hospitalar do evento inicial de SCA no estudo ERICO, a não administração de um ou mais medicamentos ocorreu em aproximadamente um terço da amostra, e a não administração/suspensão de um ou mais medicamentos ocorreu em aproximadamente metade da amostra. Os motivos para a não administração não foram descritos nos prontuários médicos dos pacientes em 64% dos casos, e os motivos para suspensão não foram descritos em 26,4% dos casos. Indivíduos com idade > 65 e aqueles com diagnóstico de angina instável apresentaram uma probabilidade mais alta de não administração de um ou mais medicamentos. Indivíduos de 65 anos de idade também apresentaram uma probabilidade mais alta de não administração/suspensão de um ou mais medicamentos.

A frequência da não administração ou suspensão do medicamento durante o tratamento de um evento de SCA foi reportada em outros cenários. Candela et al.^19^ analisaram dados de 1.134 pacientes com SCA sem supradesnivelamento de ST tratados em hospitais terciários na Espanha. Esses autores analisaram grupos de acordo com as opções de tratamento por ACTP e/ou CABG, e identificaram que, nas primeiras 24 horas, de 96,3% a 99,2% receberam aspirina, de 75,8% a 83,6% receberam heparina, e de 67,7% a 77,9% receberam clopidogrel (essa proporção pode subir para estar entre 78,3% e 99,2% entre os grupos, quando a proporção dos indivíduos que receberam prasugrel e/ou ticagrelor é acrescentada) Khedri et al.,^20^ analisaram uma amostra grande de 75.129 pacientes com SCA na Suécia usando um sistema nacional online. Nesse cenário, na alta hospitalar, aspirina não foi prescrita para 6,8% dos pacientes, betabloqueadores, para 4%, e IECA/BRA, para 31,9%. Considerando a ausência de prescrição na alta hospitalar como resultado da não administração/suspensão do medicamento durante o tratamento hospitalar, nosso estudo observou índices mais baixos de não administração ou suspensão de aspirina (3,7%) e IECA/BRA (21,5%), e índices mais altos de não administração ou suspensão de betabloqueadores (23,1%). Como razões específicas não foram exploradas no estudo de Khedri et al.,^20^ é impossível fazer inferências posteriores relacionadas às razões para tais diferenças.

Outros autores exploraram os motivos da não administração ou suspensão de medicamentos. Entretanto, diferentemente de nosso estudo, a maioria limita suas descrições a um número menor de medicamentos, ou têm o objetivo de quantificar a frequência de um motivo específico para a não administração ou suspensão. Consistente com nossos achados, Marino et al.^12^ identificaram que complicações hemorrágicas explicaram uma proporção significativa da não administração ou suspensão da aspirina, embora nossos índices de prescrição contínua durante a internação hospitalar foram ligeiramente mais altos do que seus índices de prescrição na alta hospitalar (96,3% x 93,3%). Em contraste, Bandara et al.,^21^ analisaram 81 participantes com STEMI e identificaram que 95% receberam aspirina, clopidogrel, e estatina no momento da internação hospitalar, enquanto apenas 88% receberam essas medicações no momento da alta hospitalar. Eles descrevem que o medicamento que foi suspenso com mais frequência foi a aspirina, e o motivo mais frequente foi dor epigástrica ou suspeita de hemorragia gastrointestinal. Isso contraria nossos achados, já que o uso da aspirina raramente foi suspenso durante o tratamento. Um fator de contribuição para essas diferenças é que nossa amostra não identificou nenhum indivíduo em que o tratamento com aspirina não foi prescrito ou foi retirado exclusivamente devido a dor epigástrica, já que essa não é uma contraindicação formal para o tratamento com aspirina.^22^

Marino et al.,^12^ também relataram dados sobre o uso de betabloqueadores em sua amostra. Entre 181 (30,5%) pacientes com SCA que não receberam betabloqueadores nas primeiras 24 horas em seu estudo, 39 (21,5%) tinham contraindicações identificáveis para o uso do medicamento. Embora deva haver algum cuidado com a comparação direta entre dados de pacientes nas primeiras 24 horas e dados de pacientes de todo o período de internação hospitalar, no presente estudo, os índices de não administração de betabloqueadores (15,8%) e de suspensão (4,8%) foram mais baixos, enquanto a proporção de indivíduos em que uma contraindicação poderia ser rastreada dos prontuários médicos era mais alta (56,2% e 63,4% para não administração e suspensão, respectivamente). Algumas hipóteses podem ser levantadas em relação a essas diferenças. Primeiramente, Marino et al.,^12^ incluíram indivíduos que vieram ao hospital via serviços pré-hospitalares ou que foram transferidos por ambulância de outras unidades. Como o presente estudo avaliou apenas indivíduos que vieram espontaneamente ao hospital, pode-se questionar se a proporção de indivíduos com casos mais graves (e, possivelmente, com mais contraindicações ao uso de betabloqueadores) é mais baixo em nossa amostra. Os dados de mortalidade de ambos estudos corroboraram essa hipótese. Enquanto 17,2% dos pacientes com STEMI do estudo de Marino et al.^12^ morreram antes da alta hospitalar, a mortalidade após um ano de pacientes com STEMI no estudo ERICO foi de 9,6%.^14^ Segundo, pode haver desigualdades no preenchimento dos dados do prontuário médico. Isso também é confirmado pelo estudo de Marino et al.,^12^ o choque cardiogênico foi a contraindicação mais frequente para o uso de betabloqueadores. Complicações menos graves (tais como insuficiência cardíaca descompensada e broncoespasmo) podem ter mais tendência a serem subnotificadas em comparação com as mais graves. Portanto, é possível que seus índices mais baixos de motivos definidos no prontuário médico para a não administração de betabloqueadores pode ser parcialmente causada por essa subnotificação.

Nosso estudo adotou uma estratégia conservadora em algumas análises sensíveis, excluindo indivíduos com angina instável de modelos de regressão lógica, tratando as variáveis associadas às medicações não administradas ou suspensas. Entretanto, o achado na análise principal de que os pacientes de angina instável apresentaram uma probabilidade mais alta para a não administração de um ou mais medicamentos não deve ser desconsiderado. É possível que alguns desses pacientes não tenham recebido alguns medicamentos devido a angina estável de baixo risco (caracterizado pela ausência de um histórico de doença cardiovascular, ECG normal, troponina normal, e estabilidade clínica^23^). Entretanto, algumas características do estudo ERICO coorte sugere que isso não explique totalmente nossos achados. Primeiramente, o diagnóstico de angina instável no estudo ERICO exige evidência confirmatória de SCA (por exemplo, as alterações de ECG de linha de base ou testes não invasivos positivos) ou, como alternativa, concordância de dois médicos independentes. Em segundo lugar, os indivíduos com SCA de baixo risco têm mais chances de receberem alta antecipada do atendimento de emergência. Embora essas características não impeçam a inclusão de indivíduos com angina instável de baixo risco no estudo ERICO, sua representação na amostra provavelmente estará reduzida. Portanto, nossos resultados podem, na verdade, indicar o subtratamento de indivíduos com angina instável de risco intermediário ou alto. Os achados de Breuckmann et al.,^24^ também confirmam essa interpretação. Em seu estudo, os autores analisaram dados de 1.400 pacientes com angina instável em 30 unidades de dor no peito na Alemanha e constataram que 78% dos pacientes de alto risco foram subtratados. Juntamente com nossos resultados, as evidências disponíveis que os médicos devem saber que devem evitar abordagens excessivamente conservadoras (incluindo a baixa testagem e o subtratamento) na gestão dos pacientes com angina instável.

O preenchimento dos prontuários médicos ainda é um desafio, e é importante enfatizar que uma proporção significativa dos motivos da não administração (e, em um grau mais baixo, suspensão) não pode ser recuperada dos prontuários médicos em nosso estudo. Essa informação geralmente não é relatada em outros artigos. Com base em nossos achados, pode-se questionar o fato de que é bastante possível que os prestadores de cuidado registrem situações clínicas que exigem uma mudança na prescrição (ou seja, a suspensão), mas raramente documentem as razões da introdução de medicamentos indicados por outros motivos. Como o preenchimento do prontuário é um ponto importante em relação à segurança do paciente^25^ e na tomada de decisão nos níveis individual e organizacional, nossos dados podem indicar uma oportunidade adicional de melhoria da qualidade do cuidado nesse sentido.

Nossos resultados sugerem que a idade mais alta é um marcador importante para o uso insuficiente de medicamento durante o tratamento de um evento de SCA. Isso deve ser esperado, já que a prevalência de algumas contraindicações e a incidência de efeitos adversos pode aumentar com a idade,^26^^,^^27^ embora existam evidências conflitantes.^28^ Roe et al.,^29^ analisaram dados do estudo *Targeted Platelet Inhibition to Clarify the Optimal Strategy to Medically Manage Acute Coronary Syndromes - TRILOGY ACS* (Inibição de plaquetas dirigida para esclarecer a estratégia ideal para gerenciar síndromes coronárias agudas por medicamentos) e identificaram que indivíduos de ≥ 75 anos de idade tinham um risco mais alto de sangramento importante durante 30 meses de acompanhamento, em comparação com indivíduos de <75 anos de idade (índice de risco, 2,15, 95% IC, 1,44-3,20). Embora o estudo não tenha tido o objetivo de analisar a fase hospitalar do tratamento de SCA, pode-se levantar a hipótese de que esse risco mais alto possa influenciar a decisão do médico de prescrever um medicamento específico. Entretanto, chama a atenção que, no estudo de Roe et al.,^29^ a frequência de sangramentos importantes no subgrupo de indivíduos com idade ≥75 ainda tenha sido baixa (1,8%). É plausível que, mesmo considerando-se a frequência mais alta de efeitos adversos e a contraindicação, os indivíduos com idade mais alta possivelmente estão sendo subtratados.

A presença de medicamentos não administrados ou suspensos também foi associada a um índice menor de sobrevivência após 30 dias, em nossas análises. Pode-se argumentar que esse achado reflete, pelo menos parcialmente, um efeito de subtratamento prejudicial para a sobrevivência. Entretanto, nesse contexto de um estudo de observação como o nosso, esse resultado também pode ser interpretado com cuidado. Indivíduos com doenças mais graves podem ter uma probabilidade maior de contraindicação de terapia médica. Dessa forma, as diferenças na mortalidade em curto prazo entre os grupos também podem ser influenciadas por desigualdades nas características da linha de base ou no curso da doença. A proporção baixa de indivíduos que morreram nos primeiros 30 dias (5,0%) também limita a força das conclusões dessa análise.

Nosso estudo tem alguns pontos fortes. Poucos estudos prévios apresentam uma descrição completa das razões da não administração e da suspensão dos medicamentos usados durante um evento de SCA. Especificamente, quando esses dados são apresentados, eles se limitam a um medicamento ou a um pequeno subgrupo de medicamentos. A amostra do estudo ERICO^13^^,^^14^ foi derivada de um hospital comunitário, um cenário que frequentemente não tem representação adequada em estudo coorte de SCA. Como esse estudo usou uma análise completa dos prontuários médicos, foi possível identificar as razões da não administração e da suspensão de medicamentos mesmo quando não foram explicitamente informadas nos diagnósticos dos pacientes. Nosso estudo deve ser interpretado dentro de seu contexto. Como esse é um estudo unicêntrico, conduzido em um hospital comunitário, as conclusões podem se aplicar a contextos similares aos nossos. Os dados de tratamento foram coletados na linha de base do estudo ERICO, e as alterações no cenário do estudo desde então, poderiam, potencialmente, alterar nossos achados. Entretanto, os autores acreditam que não foram feitas alterações significativas no cenário do estudo de forma a considerar que nossos achados não são mais válidos. Mesmo que fosse o caso, nossas descrições dos motivos da não administração e da suspensão dos medicamentos, a qualidade comparativa do preenchimento dos prontuários médicos (entre medicamentos não administrados e suspensos), e o subtratamento de indivíduos mais velhos são aplicáveis, principalmente, em outros cenários. Os motivos para a não administração e a suspensão não foram descritos nos prontuários médicos em 64,0% e 26,4% dos casos, respectivamente. Como discutido acima, o preenchimento do prontuário médico no atendimento de emergência raramente é descrito em artigos. Os dados faltando em nosso estudo é comparável à descrição encontrada no estudo de Marino et al.,^12^ Por outro lado, em comparação com centros terciários, os pacientes em hospitais comunitários (como o nosso) têm menos doenças graves e comorbidades. Pode-se considerar que existe uma tendência maior de não relatar contraindicações mais leves, e, portanto, isso pode se refletir na frequência relativa dos motivos da não administração ou suspensão de medicamentos em nossa amostra. Não pudemos recuperar dados de aproximadamente um quinto dos participantes potencialmente elegíveis. Devido ao desenho e aos objetivos deste estudo, apenas os indivíduos cujos dados de internação estavam completos puderam ser incluídos. Algumas dessas perdas se deveram a transferências para outros hospitais para tratamento especializado (ACTP ou cirurgia), e é possível que o subconjunto de pacientes esteja sub-representado. O ERICO é um estudo de observação e não influencia o protocolo de tratamento médico. Portanto, a decisão de não administrar ou suspender medicamentos ficava a critério do médico da emergência. Por último, como a maioria das informações do prontuário médico estava em arquivos físicos (não eletrônicos), nossos resultados para registros em prontuários médicos em relação aos motivos para não administração e/ou suspensão de medicamentos pode não ser transponível para cenários que usam principalmente registros médicos eletrônicos.

## Conclusões

Neste estudo ERICO, a não administração ou a suspensão de um ou mais medicamentos ocorreu em 51,2% da amostra. Indivíduos com 65 anos ou mais e aqueles com diagnóstico de angina instável apresentaram uma probabilidade mais alta de não administração de um ou mais medicamentos. O registro adequado em prontuários médicos ainda é um desafio e pode significar uma oportunidade adicional de melhoria da qualidade da assistência.
