# Supplementary material for: Frequência e Motivos para a não Administração e Suspensão de Medicamentos durante um Evento de Síndrome Coronariana Aguda. Estudo ERICO
Source: Arq Bras Cardiol. 2020 Nov 1;115(5):830–9. [Article in Portuguese] doi: 10.36660/abc.20190317 (PMC8452196; doi:10.36660/abc.20190317)
Supplement: Translation

# Translation: Frequency and Reasons for Non-Administration and Suspension of Drugs During an Acute Coronary Syndrome Event. The ERICO Study

**Authors:** Rafael C. O. Santos, Isabela M. Bensenor, Alessandra C. Goulart, Paulo A. Lotufo, Itamar S. Santos

**Affiliations:** 1 São PauloSP Brazil Universidade de São Paulo - Centro de Pesquisa Clínica e Epidemiológica do Hospital Universitário da USP, São Paulo, SP - Brazil; 2 São PauloSP Brazil Universidade de São Paulo - Departamento de Clínica Médica da Faculdade de Medicina da USP, São Paulo, SP - Brazil

**Keywords:** Acute Coronary Syndrome/mortality, Withholding Treatment /drug therapy, Morbidity, Health Care (Public Health)

## Abstract

**Background:**

Few studies have discussed the reasons for pharmacological undertreatment of Acute Coronary Syndrome (ACS).

**Objectives:**

To determine the frequency and reasons for the non-administration and suspension of medications during in-hospital treatments of ACS in the Strategy of Registry of Acute Coronary Syndrome (ERICO) study.

**Methods:**

The present study analyzed the medical charts of the 563 participants in the ERICO study to evaluate the frequency and reasons for the non-administration and/or suspension of medications. Logistic regression models were built to analyze if sex, age ≥65 years of age, educational level, or ACS subtype were associated with (a) the non-administration of ≥1 medications; and (b) the non-administration or suspension of ≥1 medications. The significance level was set at 5%.

**Results:**

This study's sample included 58.1% males, with a median of 62 years of age. In 183 (32.5%) participants, ≥1 medications were not administered, while in 288 (51.2%), ≥1 medications were not administered or were suspended. The most common reasons were the risk of bleeding (aspirin, clopidogrel, and heparin), heart failure (beta blockers), and hypotension (angiotensin-converting enzyme inhibitors and angiotensin receptor blockers). Individuals aged ≥65 (odds ratio [OR]:1.51; 95% confidence interval [95% CI]:1.05-2.19) and those with unstable angina (OR:1.72; 95% CI:1.07-2.75) showed a higher probability for the non-administration of ≥1 medication. Considering only patients with myocardial infarction, being ≥65 years of age was associated with both the non-administration and the non-administration or suspension of ≥1 medication.

**Conclusions:**

Non-administration or suspension of ≥1 medication proved to be common in this ERICO study. Individuals of ≥65 years of age or with unstable angina showed a higher probability of the non-administration of ≥1 medication and may be undertreated in this scenario. (Arq Bras Cardiol. 2020; 115(5):830-839)

## Introduction

Coronary artery disease (CAD) continues to be the leading cause of mortality and disability-adjusted life years worldwide, including Brazil.^1^^–^^4^ Appropriate and timely treatment may reduce morbidity and mortality.^5^ There is evidence that the quality of pharmacological treatment in the hospital phase of an acute coronary syndrome (ACS) event, defined by the early administration of guideline-oriented medications, is associated with in-hospital survival^6^ and six-month survival.^7^

In the largest Brazilian study reporting the frequency of guideline-oriented medication prescriptions in hospitalized ACS patients to date, Wang et al.^8^ evaluated data from 2,453 individuals with ACS from 65 Brazilian hospitals (approximately 90% tertiary hospitals) in the study of the Acute Coronary Care Evaluation of Practice (ACCEPT) Registry from August 2010 to December 2011. Among the drugs analyzed in their study, aspirin was the most commonly prescribed drug in the first 24 hours (97.6%). Statins also presented a high frequency of prescription (90.6%).

Few studies have discussed the reasons for ACS undertreatment. This is especially important as the mean age of ACS patients is on the rise. Adverse effects and contraindications are more frequent^9^ in older individuals, contributing to their associated higher morbidity and mortality.^10^^,^^11^ Marino et al.^12^ evaluated 583 individuals diagnosed with ACS in six emergency hospitals in Montes Claros. In the first 24 hours of treatment, the use of medications for ACS treatment varied from 63.8% (heparins) to 96.6% (aspirin). Of 181 patients (31.0% of their sample) who did not receive beta blockers within 24 hours, 39 (21.5%) presented identifiable contraindications. No other descriptions of the reasons for under treatment during the first 24 hours were reported.

The present article seeks to analyze data from ACS events, which led to the enrollment of 563 participants in the Strategy of Registry of Acute Coronary Syndrome (ERICO) study, a prospective study that is still ongoing at the University Hospital of the University of São Paulo (HU-USP in Portuguese). Our team aimed to determine the frequency of use, along with the reasons for the non-administration and suspension of medications used during the in-hospital treatment of an ACS event and their associated factors.

## Methods

### ERICO Study design

The design of the ERICO study has been described in detail elsewhere.^13^^,^^14^ Briefly, ERICO is a prospective observational study of 1,085 individuals admitted to the HU-USP due to an ACS event between February 2009 and December 2013. HU-USP is a community hospital in Butantã, a district in the city of Sao Paulo, Brazil, with an estimated population of 428,000 inhabitants in 2010 and marked socioeconomic inequalities.

For participation in the ERICO study, participants were required to meet the diagnostic criteria for ST-elevation myocardial infarction (STEMI), non-ST-elevation myocardial infarction (NSTEMI) or unstable angina (UA). For a diagnosis of myocardial infarction (MI), both of the following criteria must be present: (I) Symptoms consistent with cardiac ischemia within 24 hours of hospital admission and (II) Troponin I levels above the 99^th^ percentile with a test-specific coefficient of variation <10%. The STEMI diagnosis requires both of the following criteria: (I) Criteria for MI diagnosis and (II) One of the following: (a) persistent ST segment elevation of ≥ 1 mm in two contiguous electrocardiographic leads or (b) the presence of a new or presumably new left bundle branch block. For the NSTEMI diagnosis, participants must present: (I) Criteria for MI diagnosis and (II) Absence of criteria for STEMI diagnosis. For UA diagnosis, all of the following three criteria must be fulfilled: (I) Symptoms consistent with cardiac ischemia 24 hours prior to hospital admission, (II) Absence of MI criteria, and (III) At least one of the following: (a) history of coronary artery disease; (b) positive coronary disease stratification test (invasive or noninvasive); (c) transient ST segment changes ≥ 0.5 mm in two contiguous leads, new T-wave inversion of ≥ 1 mm and/or pseudo-normalization of previously inverted T waves; (d) troponin I > 0.4 ng/ml; or (e) diagnostic agreement of two independent physicians. Non-ST elevation acute coronary syndrome (NSTEACS) is a common term that encompasses NSTEMI and UA.

At baseline, trained interviewers obtained data on sociodemographic and cardiovascular risk factors, as well as previous medications. During the in-hospital phase, all subjects were treated at the discretion of the hospital staff, with standard procedures and with no influence from the study protocol. Long-term follow-up is currently ongoing, with annual telephone contacts.

### ERICO-APS study design

The present paper is an analysis of an ancillary ERICO study (Strategy of Registry of Acute Coronary Syndrome – Primary Health Care; ERICO-APS study). Further detail about the ERICO-APS study can be found in a previous publication.^15^ ERICO-APS aims to study determinants of quality of care and mortality, with a special focus on the unit of first contact (primary care or hospital) during the index ACS event. ERICO-APS comprises 130 participants for whom a primary care facility was the unit of first contact during the index event, and 700 participants who came directly to the hospital, all enrolled in the main study from February 2009 to December 2012.

### Study sample

In our analyses, 700 ERICO-APS participants who came directly to the hospital were eligible. This study excluded 44 (6.3%) participants whose medical charts could not be retrieved and 93 (13.3%) whose medical chart data were incomplete (for example, due to transfer to other hospitals). Our final sample consisted of 563 ERICO-APS participants.

### Study variables

Hypertension, diabetes, dyslipidemia, and previous coronary artery disease (CAD) diagnoses were defined by self-report. Smoking status was classified as never, past, or current smoker. The educational level was self-reported and classified as no formal education, 1 to 7 years of formal education, and ≥ 8 years of formal education. In some of the analyses, age was categorized using a cutoff of 65 years.

Medical charts and prescriptions were reviewed in order to analyze the frequency of administration, reasons for non-administration, and reasons for the suspension of the following medications: aspirin, clopidogrel, heparins, beta blockers, and angiotensin-converting enzyme inhibitors and angiotensin receptor blockers (ACEI/ARB). The frequency of the administration for statins, nitrates, and morphine was also analyzed.

“Non-administration” was defined as the non-prescription of medications from admission to discharge. “Suspension” was defined as the withdrawal of drugs initially prescribed during the hospitalization period. One exception was the withdrawal of the heparin prescription after the eighth day of hospitalization.^16^ The reasons were separated by pharmacological class: (a) aspirin: allergy, bleeding or risk of bleeding, and revascularization surgery; (b) clopidogrel: bleeding or risk of bleeding and coronary artery bypass; (c) heparin: bleeding or risk of bleeding, revascularization surgery, low risk acute coronary syndrome, and coronary angiography; (d) beta blockers: bronchospasm, bradycardia, shock/hypotension, decompensated heart failure, and non-invasive testing for ischemia; and (e) ACEI/ARB: chronic renal failure (CRF), shock/hypotension, acute renal failure (ARF), and hyperkalemia.

These reasons are described in supplementary table 1, along with the most frequently prescribed drugs for each pharmacological class. The non-administration (or suspension) of any medication was defined as the non-administration (or suspension) of one or more of the following: aspirin, clopidogrel, heparin, beta blockers, statins, and/or ACE inhibitors/ARB.

**Table 1:** Baseline characteristics of the study sample

		STEMI (N=162)	NSTEMI (N=232)	UA (N=169)	Total (N=563)
Age (years; median [IQR])		59.0 [50.0 - 68.0]	64.0 [53.8 - 74.0]	62.0 [53.0 - 73.0]	62.0 [52.0 - 72.0]
Male sex		106 (65.4%)	140 (60.3%)	81 (47.9%)	327 (58.1%)
Educational level					
	No formal education	16 (9.9%)	24 (10.3%)	22 (13.0%)	62 (36.7%)
	1 to 7 years	69 (42.9%)	107 (46.1%)	62 (11.0%)	238 (42.3%)
	≥ 8 years	76 (47.2%)	101 (43.5%)	85 (50.3%)	262 (46.6%)
Hypertension		101 (64.3%)	174 (76.0%)	146 (89.0%)	421 (76.5%)
Diabetes		49 (31.4%)	99 (42.9%)	67 (41.4%)	215 (39.2%)
Dyslipidemia		66 (50.0%)	113 (53.3%)	83 (57.2%)	262 (53.6%)
Sedentarism		98 (66.2%)	156 (70.6%)	115 (73.7%)	369 (70.3%)
Smoking status					
Never		37 (23.7%)	69 (31.7%)	60 (38.5%)	166 (31.3%)
Past		57 (36.5%)	81 (37.2%)	62 (39.7%)	200 (37.7%)
Current		62 (39.7%)	68 (31.2%)	34 (21.8%)	164 (30.9%)
Previous CAD		25 (16.9%)	50 (23.3%)	75 (49.3%)	150 (29.1%)

IQR: interquartile range; STEMI: ST-segment elevation myocardial infarction; NSTEMI: non-ST-segment elevation myocardial infarction; UA: unstable angina; CAD: coronary artery disease.

When the reason for drug non-administration or suspension was noted in the medical charts, this information was retrieved and classified according to its explicit reason. Whenever these reasons for non-administration or suspension were not explicit, a doctor and a pharmacist from the study reviewed the medical chart to verify whether any of the described reasons were implicit. Therefore, the reasons for non-administration or suspension were classified as “not described”, “implicit”, or “explicit”.

Vital status was assessed by telephone interview 30 days after the index event, according to ERICO study protocol.^14^^,^^17^ Official death records were obtained with the collaboration of the municipal and state's health offices whenever it was verified that the participant had died or if the patient could not be contacted at that time.

### Ethical considerations

The study protocol was in accordance with the Declaration of Helsinki. The hospital's institutional review board approved the research protocol (Ethical Committee Approval 866/08). Written informed consent was obtained from all ACS patients admitted to the hospital who agreed to participate in this study, and each subject received a copy of the informed consent form.

### Statistical analysis

Categorical variables are presented as absolute counts and proportions, and compared using chi-squared tests. Due to its non-normal distribution (evaluated by density plots and the Shapiro-Wilk test), age is presented as a median and interquartile range and compared among groups using the Kruskal-Wallis test. This study also performed pairwise comparisons (with Holm adjustment) for age distribution in STEMI, NSTEMI, and UA groups. Crude and multiple logistic regression models were built to analyze if sex, being ≥ 65 years of age, educational level, or ACS subtype were associated with (a) the non-administration of any medication and (b) the non-administration or suspension of any medication. As sensitivity analyses, these models were repeated: (a) excluding the non-administration/suspensions due to the scheduled percutaneous transluminal coronary angioplasty (PTCA) and/or coronary artery bypass graft (CABG) and (b) excluding those with unstable angina, as some medications may not have been prescribed due to low-risk ACS. Kaplan-Meier curves and the log-rank test were used to determine if 30-day survival was associated with ≥ 1 non-administered or suspended medications. The significance level was set at 5%. The R software, version 3.2.0, was used to conduct these analyses.^18^

## Results

Table 1 shows the baseline characteristics of the study sample, according to the ACS subtype. This study's sample had a predominance of males (n=327; 58.1%), with a median of 62 years of age. Individuals with STEMI had a lower age compared to participants with NSTEMI (p=0.002) and UA (p=0.024). Age distribution in participants with NSTEMI and UA is not significantly different (p=0.35). Hypertension (n=421; 76.5%) and sedentarism (n=369; 70.3%) were the most frequent cardiovascular risk factors in the sample. Only 150 (29.1%) of the participants had a CAD diagnosis prior to the ACS event that led to the enrollment in the ERICO study.

Table 2 shows the frequency of the administration of aspirin, clopidogrel, heparins, statins, beta blockers, ACEI or BRA, nitrates, and morphine during in-hospital treatment. Considering the main medications in ACS treatment (aspirin, clopidogrel, heparin, beta blockers, statins, and/or ACE inhibitors/ARB), this study identified 183 (32.5%) participants in whom one or more medications were not administered. Nitrate use was similar according to ACS subtype (p=0.32) and, as expected, morphine administration was more frequent in the participants with a STEMI diagnosis (p<0.001). In 288 (51.2%) participants, this study observed the non-administration or suspension of one or more of the main medications during in-hospital treatment.

**Table 2:** Administration of guideline-oriented medications during in-hospital treatment

Drug	STEMI	NSTEMI	UA	Total
Aspirin	158 (97.5%)	229 (98.7%)	165 (97.6%)	552 (98.0%)
Clopidogrel	159 (98.1%)	226 (97.4%)	158 (93.5%)	543 (96.4%)
Heparin	153 (94.4%)	228 (98.3%)	160 (94.7%)	541 (96.1%)
Statins	152 (93.8%)	217 (93.5%)	147 (87.0%)	516 (91.7%)
Beta-blockers	138 (85.2%)	194 (83.6%)	142 (84.0%)	474 (84.2%)
ACEI/ARB	136 (84.0%)	201 (86.6%)	132 (78.1%)	469 (83.3%)
Nitrate	95 (58.6%)	119 (51.3%)	95 (56.2%)	309 (54.9%)
Morphine	37 (22.8%)	30 (12.9%)	9 (5.3%)	76 (13.5%)

STEMI: ST-segment elevation myocardial infarction; NSTEMI: non-ST-segment elevation myocardial infarction; UA: unstable angina; ACEI/ARB: angiotensin-converting enzyme inhibitors or angiotensin receptor blockers.

Table 3 presents the reasons for the non-administration or suspension of aspirin, clopidogrel, heparin, beta blockers, and ACEI/ARB. It was observed that the non-administration or suspension of aspirin, clopidogrel, and heparin is a rare event, usually linked to an increased risk of bleeding. The most frequent reason for the non-administration of beta blockers were decompensated heart failure and shock/hypotension. Heart failure was also the most frequent reason for beta blocker suspension. Shock/hypotension was the most frequent reason for the non-administration and suspension of ACEI/ARB. Supplementary Table 2 reports the frequencies for the presence of reasons for the non-administration/suspension of medications in the medical charts. It was observed that the reasons for non-administration were not described in the medical charts in 64.0% of the cases, and the reasons for suspension were not described in 26.4%.

**Table 3:** Causes for the non-administration or suspension of medications in the sample

Drug	Cause	Non-administration	Suspension
Aspirin	Allergy	4	0
	Bleeding or risk of bleeding	1	5
	Coronary artery bypass graft	0	5
	Total	5	10
Clopidogrel	Bleeding or risk of bleeding	1	15
	Coronary artery bypass graft	1	2
	Coronary angiography	0	22
	Total	2	39
Heparin	Bleeding or risk of bleeding	2	7
	Coronary artery bypass graft	0	4
	Coronary angiography	0	35
	Low-risk acute coronary syndrome	2	0
	Total	4	46
Beta-blockers	Decompensated heart failure	16	11
	Bronchospasm	14	5
	Shock / hypotension	14	5
	Bradycardia	6	4
	Non-invasive testing for ischemia	0	1
ACEI/ARB	Shock / hypotension	14	9
	Chronic renal failure	6	0
	Hyperkalemia	3	5
	Acute renal failure	1	7
	Total	24	21

ACEI/ARB: angiotensin-converting enzyme inhibitors and angiotensin receptor blockers.

Table 4 shows the odds ratios (from multiple models) for the non-administration and non-administration/suspension of one or more medications (aspirin, clopidogrel, heparin, statins, and/or ACE inhibitors/ARB), associated with age, sex, educational level, and ACS subtype. Analyzing the entire sample, individuals aged 65 or older (p=0.027) and those with unstable angina (p=0.025) presented a higher probability for the non-administration of one or more medications. When individuals with unstable angina were excluded, being ≥ 65 years of age was associated with either the non-administration (p=0.023) or the non-administration/suspension (p=0.035) of one or more medications. In this subsample, individuals with STEMI or NSTEMI presented a similar probability for the non-administration (p=0.73) or the non-administration/suspension (p=0.85) of one or more medications.

**Table 4:** Odds ratios (95% CI) from multiple models for the association between non-administration and non-administration or suspension with age, sex, educational level, and ACS subtype

		All ACS subtypes		Excluding participants with UA	
		Non-administration	Non-administration or suspension	Non-administration	Non-administration or suspension
Male sex		0.96 (0.67 - 1.39)	0.88 (0.62 - 1.24)	0.98 (0.62 - 1.55)	0.93 (0.61 - 1.41)
Age ≥ 65 years		1.51 (1.05 - 2.19)	1.36 (0.96 - 1.92)	1.69 (1.07 - 2.67)	1.57 (1.03 - 2.40)
Educational level					
	No formal education	0.58 (0.31 - 1.11)	0.58 (0.32 - 1.03)	0.58 (0.25 - 1.33)	0.55 (0.26 - 1.13)
	1 to 7 years	0.90 (0.61 - 1.31)	1.10 (0.77 - 1.58)	0.95 (0.60 - 1.51)	1.16 (0.76 - 1.76)
	≥ 8 years	1.0 (Reference)	1.0 (Reference)	1.0 (Reference)	1.0 (Reference)
ACS subtype					
	STEMI	1.0 (Reference)	1.0 (Reference)	1.0 (Reference)	1.0 (Reference)
	NSTEMI	1.10 (0.70 - 1.73)	0.98 (0.65 - 1.48)	1.08 (0.69 - 1.71)	0.96 (0.64 - 1.45)
	UA	**1.72 (1.07 - 2.75)**	1.23 (0.79 - 1.91)	-	-

p<0.05 in bold. ACS: Acute coronary syndrome; STEMI: ST-segment elevation myocardial infarction; NSTEMI: non-ST-segment elevation myocardial infarction; UA: unstable angina.

Sensitivity analyses, considering that participants with programmed PTCA and CABG did not qualify as a reason for the non-administration and/or suspension of clopidogrel and heparins (Supplementary Table 3), led to similar conclusions, except for a significant association between being ≥ 65 years of age and the non-administration/suspension of one or more medications (Odds ratio: 1.44; 95% CI: 1.02 – 2.04). Supplementary Tables 4 and 5 show the results obtained from the crude models.

At 30 days, eight (2.9%) individuals who had all medications administered without suspension and 20 (6.9%) individuals with one or more non-administered or suspended medications had died (Figure 1). Survival at 30-days was significantly associated with the presence of one or more non-administered or suspended medications (p=0.03).

**Figure 1:** Survival at 30 days for individuals who (a) had all medications administered without suspension and (b) had one or more non-administered or suspended medications.

## Discussion

The present study observed that, during the in-hospital treatment of the index ACS event in the ERICO study, the non-administration of one or more medications occurred in approximately one-third of the sample, and the non-administration/suspension of one or more medications occurred in approximately one half of the sample. The reasons for non-administration were not described in the patients' medical charts in 64.0% of the cases, and the reasons for suspension were not described in 26.4%. Individuals aged > 65 and those with a diagnosis of unstable angina presented a higher probability of the non-administration of one or more medications. Individuals of 65 years of age also presented a higher probability of the non-administration/suspension of one or more medications.

The frequency of the non-administration or suspension of medication during the treatment of an ACS event has been reported in other settings. Candela et al.^19^ analyzed data from 1,134 patients with non-ST segment elevation ACS treated in tertiary hospitals in Spain. These authors analyzed groups according to PTCA and/or CABG treatment options, and found that within the first 24 hours, 96.3% to 99.2% received aspirin, 75.8% to 83.6% received heparin and 67.7% to 77.9% received clopidogrel (this proportion may rise to 78.3% to 99.2% among groups, when the proportion of individuals receiving prasugrel and/or ticagrelor are added). Khedri et al.^20^ analyzed a large sample of 75,129 patients with ACS in Sweden using a nationwide web-based system. In that setting, upon hospital discharge, aspirin was not prescribed for 6.8% of the patients, beta blockers for 11.4%, and ACEI/ARBs for 31.9%. Considering the absence of prescription upon hospital discharge as the result of the non-administration or suspension of medication during hospital treatment, our study observed lower rates of the non-administration or suspension of aspirin (3.7%) and ACEI/ARBs (21.5%) and higher rates of the non-administration or suspension of beta blockers (23.1%). As specific reasons were not explored in Khedri et al.^20^ study, it is impossible to make further inferences concerning the reasons for those differences.

Other authors have explored the reasons for the non-administration or suspension of medication. However, unlike our study, most limit their descriptions to a smaller number of medications, or aim to quantify the frequency of a specific reason for a non-administration or suspension. Consistent with our findings, Marino et al.^12^ identified that hemorrhagic complications explained a significant proportion of the non-administration or suspension of aspirin, although our rates of uninterrupted in-hospital prescription were slightly higher than their prescription rates upon hospital discharge (96.3% vs 93.3%). By contrast, Bandara et al.^21^ analyzed 81 participants with STEMI and found that 95% received aspirin, clopidogrel, and statin upon hospital admission, while only 88% received these medications upon hospital discharge. They describe that the most common discontinued medication was aspirin, and the most frequent reason was epigastric pain or presumed gastrointestinal hemorrhage. This contrasts with our findings, as aspirin was rarely discontinued during treatment. One possible contributor to these differences is that our sample identified no individuals in whom aspirin treatment was non-prescribed or withheld due exclusively to epigastric pain, as this is not a formal contraindication to aspirin treatment.^22^

Marino et al.^12^ also reported data about beta blocker use in their sample. Among 181 (30.5%) patients with ACS who did not receive a beta blocker in the first 24 hours in their study, 39 (21.5%) had identifiable contraindications to drug use. Although there must be some caution in directly comparing 24-hour patient data with full hospital stay patient data, in the present study, rates for beta blocker non-administration (15.8%) and suspension (4.8%) were lower, while the proportion of individuals in which a contraindication could be retrieved from the medical charts was higher (56.2% and 63.4% for non-administration and suspension, respectively). Some hypotheses may be raised in relation to these differences. First, Marino et al.^12^ included individuals who came to the hospital through pre-hospital services or who were transferred by ambulance from other units. As the present study evaluated only individuals who came spontaneously to the hospital, one can speculate that the proportion of individuals with more severe cases (and, potentially, with more contraindications to beta blocker use) is lower in our sample. Mortality data from both studies corroborate this hypothesis. While 17.2% of the STEMI patients in Marino et al.^12^ study died before hospital discharge, one-year mortality for STEMI patients in the ERICO study was 9.6%.^14^ Second, there may be inequalities in the completeness of the medical chart data. This is further supported by the fact that in Marino et al.^12^ study, cardiogenic shock was the most frequent contraindication for beta blocker use. Less severe complications (such as decompensated heart failure and bronchospasm) may be more prone to under-reporting compared to more severe ones. Therefore, it is possible that their lower rates of medical chart-defined reasons for beta blocker non-administration may be partially caused by this under-reporting.

Our study adopted a conservative strategy in some sensitivity analyses, excluding individuals with unstable angina from logistic regression models addressing variables associated with non-administered or suspended medications. However, the finding in main analyses that unstable angina patients presented a higher probability for the non-administration of one or more medications should not be overlooked. It is possible that some of these patients did not receive some medications due to low-risk unstable angina (characterized by the absence of a history of cardiovascular disease, normal ECG, normal troponin, and clinical stability^23^). However, some characteristics of the ERICO cohort suggest this may not fully explain our findings. First, the diagnosis of unstable angina in ERICO requires confirmatory evidence of ACS (for example, by baseline ECG alterations or positive non-invasive testing) or, alternatively, concordance by two independent physicians. Second, individuals with low-risk ACS are more prone to receive early discharge from the emergency clinic. Although these features do not preclude the inclusion of individuals with low-risk unstable angina in the ERICO study, their representation in the sample is probably reduced. Therefore, our results may actually point to an undertreatment of individuals with intermediate- or high-risk unstable angina. The findings from Breuckmann et al.^24^ support this interpretation as well. In their study, the authors analyzed data from 1,400 patients with unstable angina in 30 chest pain units in Germany and found that 78% of the high-risk patients were undertreated. Along with our results, available evidence suggests that physicians should be aware to avoid overly conservative approaches (including undertesting and undertreating) in the management of patients with unstable angina.

The completeness of medical chart data is still challenging, and it is important to emphasize that a significant proportion of reasons for non-administration (and, to a lesser extent, suspension) could not be retrieved from medical charts in our study. This information is not usually reported in other articles. Based on our findings, one can speculate that caregivers are fairly likely to register a clinical situation requiring a change in prescription (i.e., suspension) but rarely document the reasons for not introducing an otherwise indicated medication. As medical chart completeness is an important point related to patient safety^25^ and decision-making in individual and organizational levels, our data may point to an additional opportunity to improve the quality of care in this regard.

Our results suggest that higher age is an important marker for medication underuse during the treatment of an ACS event. This is to be expected, as the prevalence of some of the contraindications and the incidence of adverse effects may increase with age,^26^^,^^27^ although conflicting evidence does exist.^28^ Roe et al.^29^ analyzed data from the Targeted Platelet Inhibition to Clarify the Optimal Strategy to Medically Manage Acute Coronary Syndromes (TRILOGY ACS) trial and found that individuals of ≥ 75 years of age had a higher risk for major bleeding during 30 months of follow-up, compared to those of <75 years of age (Hazard ratio, 2.15, 95% CI, 1.44-3.20). Although that study was not intended to analyze the in-hospital phase of ACS treatment, it can be hypothesized that this higher risk may influence the physicians' decision to prescribe a specific medication. However, it is noteworthy that in Roe et al.'s^29^ study, the frequency of major bleeding in the subgroup of individuals of ≥75 years of age was still low (1.8%). It is plausible that, even considering a higher frequency of adverse effects and contraindications, individuals with higher ages are possibly being undertreated.

The presence of non-administered or suspended medications was also associated with poorer 30-day survival in our analyses. It is arguable that this finding reflects, at least partially, a detrimental effect of undertreatment on survival. However, in the context of an observational study like ours, this result must also be interpreted with caution. Individuals with more severe disease may be more prone to have contraindications to medical therapy. Therefore, differences in short-term mortality between groups may also be influenced by inequalities in baseline characteristics or in the course of the disease. The low proportion of individuals who died in the first 30 days (5.0%) also limits the strength of conclusions from this analysis.

Our study has some strengths. Few previous studies present a thorough description of reasons for the non-administration and suspension of medications used during an ACS event. In particular, when these data are presented, they are limited to one or a small subset of medications. The ERICO study sample^13^^,^^14^ is derived from a community hospital, a setting frequently under-represented in ACS cohorts. As this study used a complete review of medical charts, it was able to identify the reasons for the non-administration and suspension of medications even when they were not explicitly stated in patient diagnoses. Our study should be interpreted within its context. As this is a single-center study conducted in a community hospital, conclusions may be applicable only in contexts similar to ours. Treatment data in our article were collected at the ERICO study baseline, and alterations in the study setting since then could, potentially, change our findings. However, the authors believe that no substantial change in the study setting was made in such a ways as to consider our findings to be no longer valid. Even if this were the case, our descriptions of the causes for the non-administration and suspension of medication, comparative quality of medical chart completeness (between non-administered and suspended medications), and the undertreatment of older individuals are mostly applicable in other settings. Reasons for the non-administration and suspension were not described in the medical charts in 64.0% and 26.4% of the cases, respectively. As discussed above, medical chart completeness in the emergency clinic is rarely described in articles. Missing chart data in our study is comparable to the description found in Marino et al.'s study^12^. On the other hand, in comparison to tertiary centers, patients in community hospitals (like ours) have less severe disease and comorbidities. It is reasonable to consider that milder contraindications are more prone to underreporting, and, therefore, this may reflect on the relative frequency of causes for the non-administration or suspension of medications in our sample. We could not retrieve complete data from approximately one-fifth of the potentially eligible participants. Due to the design and objectives of this study, only individuals with complete inpatient data could be included. Some of these losses were due to transfers to other hospitals for specialized treatment (PTCA or surgery), and it is possible that this subset of patients is under-represented in our sample. ERICO is an observational study and does not influence medical treatment by protocol. Therefore, the decision not to administer, or to suspend medications, was under the discretion of the emergency ward's physician. Finally, as most of the medical chart information was in physical (non-electronic) files, our results for medical chart records regarding the reasons for the non-administration and/or suspension of medications may not be transposable to settings using mainly electronic medical records.

## Conclusions

In this ERICO study, the non-administration or suspension of one or more medications occurred in 51.2% of the sample. Individuals aged 65 or older and those with unstable angina diagnosis presented a higher probability of the non-administration of one or more medications. Adequate medical chart registry is still challenging and may present an additional opportunity to improve the quality of care.
